# Sensor-Driven Short-Term Forecasting on the Metropolitan LA Traffic Dataset: A Comparative Study for Multi-Step Prediction

**DOI:** 10.3390/s26123917

**Published:** 2026-06-20

**Authors:** Bowen Dong, Xinyu Zhang, Weiyan Zhu, Lingmin Hou, Chaoya Yan, Yifan Feng, Lixing Lin

**Affiliations:** 1 School of Electrical Automation and Information Engineering, Tianjin University, Tianjin 300072, China; 2 Department of Computer Science, Rochester Institute of Technology, Rochester, NY 14623, USA; 3 Meta Platforms Inc., Menlo Park, CA 94025, USA; 4 Department of Computer Science, Rutgers University, New Brunswick, NJ 08901, USA; 5 Department of Computer Science and Engineering, Santa Clara University, Santa Clara, CA 95053, USA; 6 Graduate School of Arts and Science, Yale University, New Haven, CT 06520, USA

**Keywords:** short-term traffic forecasting, intelligent transportation systems, METR-LA, traffic sensor networks, spatiotemporal prediction, Transformer, graph-based forecasting

## Abstract

Short-term traffic forecasting is a critical component of intelligent transportation systems. While deep learning architectures for this task have proliferated rapidly, the sensor-level data characteristics—zero-value prevalence, distributional heterogeneity, and cross-sensor correlation structure—that drive architecture-specific failure modes remain insufficiently understood, and their implications for evidence-based model selection in real deployments have not been systematically addressed. This study addresses that question through a sensor-network diagnostic framework applied to the METR-LA dataset (Metropolitan Los Angeles; 207 inductive loop detectors, 5-min resolution). The framework integrates systematic characterization of sensor data properties, a controlled benchmark of four representative architectures—Transformer, Spatio-Temporal Graph Convolutional Network (STGCN), Diffusion Convolutional Recurrent Neural Network (DCRNN), and Gated Temporal Convolutional Network (Gated TCN)—under a unified 12→3 prediction setting, and a novel per-sensor regression analysis that quantitatively links zero-value ratios to model-specific prediction errors across all 207 sensors. Building on these findings, this study further proposes Graph-Enhanced Transformer (GETFormer), a lightweight hybrid architecture that augments the Transformer with a single-hop Graph Convolutional Network (GCN) layer and a gated residual fusion module. The diagnostic findings and condition-dependent model-selection guidelines provide an empirically grounded foundation for principled hybrid architecture development in urban traffic sensing.

## 1. Introduction

### 1.1. Research Background and Motivation

Short-term traffic forecasting has become a core task in intelligent transportation systems, since reliable prediction of near-future traffic states directly supports congestion management, route guidance, travel time estimation, and real-time traffic control [1]. As urban road networks continue to expand and traffic demand intensifies, the ability to predict short-horizon traffic conditions with sufficient accuracy is both theoretically and practically important [2]. In operational settings, even modest improvements in forecasting reliability can enhance the efficiency and responsiveness of network-level traffic management [3].

The rapid deployment of urban sensing infrastructure has fundamentally reshaped traffic forecasting research. Roadside monitoring devices, particularly loop detectors, continuously record traffic variables such as speed, flow, and occupancy, generating large-scale spatiotemporal observations over extended periods [4]. These sensing systems no longer provide isolated measurements; rather, they form distributed urban observation networks in which each sensor captures a localized traffic state while the entire network reflects spatiotemporal traffic dynamics propagating and interacting across space and time. Under such conditions, traffic forecasting should be regarded not merely as a conventional time-series problem, but as a sensor-driven inference task that depends on the joint modeling of temporal evolution and inter-sensor dependency.

Public benchmark datasets have played an important role in advancing this area. Among them, METR-LA (Metropolitan Los Angeles; a public traffic-speed dataset comprising 207 inductive loop detectors deployed on highways in Los Angeles County, CA, USA) is widely adopted for evaluating short-term traffic forecasting models under realistic multi-sensor conditions [5]. The dataset contains speed observations collected at 5 min intervals, which makes it particularly suitable for examining urban traffic dynamics at a fine temporal scale. In the present study, the forecasting task is formulated as a 12→3 setting, where the previous 12 time steps are used to predict traffic speeds over the subsequent 3 time steps. Such a formulation corresponds to a practical short-horizon prediction scenario and provides a controlled setting for comparing different modeling strategies.

Recent years have witnessed substantial progress in deep learning-based traffic forecasting [6]. Recurrent architectures, temporal convolutional models, graph neural networks, and attention-based methods have each demonstrated notable capacity to capture nonlinear temporal variation and spatial interaction [7]. Even so, performance advantages reported in the literature often depend strongly on experimental conditions, including preprocessing design, graph construction strategy, training budget, and evaluation protocol. This situation creates a clear need for unified empirical studies that can reveal how different forecasting paradigms behave when trained and assessed under consistent conditions. Against this background, the present work examines, under a unified experimental setting, how representative forecasting architectures perform on short-horizon traffic speed prediction for the Metropolitan LA (METR-LA) dataset, and what these results reveal from the perspective of urban sensor networks. The study formulates a 12→3 multi-step forecasting task and evaluates four representative architectures—Transformer, STGCN, DCRNN, and Gated TCN—under consistent preprocessing, data partitioning, training epochs, and evaluation criteria, with the goal of clarifying how temporal modeling capacity, graph representation, and sensor data characteristics jointly shape forecasting effectiveness.

### 1.2. Main Contributions

The main contributions of this study are summarized as follows.

(1) A reproducible benchmarking framework is established in which sensor-level data characterization, distance-threshold graph construction, and multi-step forecasting evaluation are integrated under strictly identical conditions across all compared architectures, removing the confounds that commonly arise from inconsistent preprocessing or unequal training budgets.
(2) Three salient data-level properties of the METR-LA sensing network are systematically characterized through exploratory analysis: left-skewed speed distributions with non-negligible zero-value behavior, structured intraday and weekly temporal regularity, and heterogeneous but spatially correlated cross-sensor dynamics. These properties are explicitly linked to subsequent modeling decisions and performance interpretation.
(3) A lightweight hybrid architecture, Graph-Enhanced Transformer (GETFormer), is proposed and evaluated. GETFormer augments a Transformer temporal encoder with a single-hop graph convolution and a gated residual fusion module, achieving an MAE of 3.34 mph (a 5.11% relative reduction over the Transformer baseline) while remaining computationally lightweight. Ablation and sensor-group analyses confirm that both the graph convolution and the gating mechanism contribute independently and that the improvement is largest for sensors with high zero-value rates.
(4) A sensor-level diagnostic analysis systematically links zero-value prevalence, distributional heterogeneity, and graph construction quality to specific model failure modes, yielding four concrete, condition-dependent model-selection guidelines for practitioners deploying traffic forecasting systems in sensor-rich urban environments.

### 1.3. Organization of the Paper

The remainder of this paper is organized as follows.

Section 2 reviews related studies on traffic sensing, short-term traffic forecasting, and spatiotemporal deep learning.

Section 3 presents the dataset, exploratory analysis, preprocessing strategy, graph construction, forecasting models, and evaluation protocol.

Section 4 reports the experimental results and provides a comparative analysis of model performance.

Section 5 discusses the main findings, practical implications, limitations, and future research directions, and concludes the paper.

## 2. Literature Review

### 2.1. Traffic Sensing and Urban Traffic Monitoring

The development of short-term traffic forecasting is closely linked to the advancement of urban traffic sensing infrastructure [8]. Modern intelligent transportation systems rely on distributed monitoring devices, such as loop detectors, roadside sensors, cameras, and connected sensing platforms, to capture the dynamic states of urban road networks [9]. Among these sensing modalities, loop detectors remain one of the most widely used data sources in traffic forecasting studies because they provide continuous and relatively stable measurements of traffic speed, flow, and occupancy over long periods.

From an application perspective, traffic sensing systems serve not only as data acquisition tools but also as the observational foundation for traffic state estimation and prediction [10]. Since traffic evolution is inherently spatially interconnected, measurements collected by individual sensors must be interpreted within a broader network context [11]. Sensors located on adjacent road segments, within the same corridor, or near bottlenecks often exhibit correlated variations, reflecting the propagation of congestion and the coupling of local traffic conditions. This characteristic makes traffic forecasting fundamentally different from conventional single-series prediction tasks and highlights the importance of exploiting inter-sensor dependency in model design.

At the same time, urban sensing data present several practical challenges. Traffic measurements are often affected by missing observations, zero readings, outliers, and noise arising from hardware limitations, communication failures, or transient operational anomalies [12]. In addition, the statistical properties of traffic variables may vary considerably across locations and time periods due to heterogeneous road functions, recurrent commuting patterns, and irregular disturbances [13]. These issues imply that traffic forecasting performance depends not only on predictive architectures but also on the quality of sensor data preprocessing, representation, and structural interpretation. As a consequence, sensor-driven traffic prediction studies increasingly emphasize the full analytical pipeline, spanning data cleaning, exploratory analysis, spatial relation modeling, and forecasting evaluation.

### 2.2. Short-Term Traffic Forecasting Methods

Early studies on short-term traffic forecasting were mainly based on statistical and shallow machine learning methods. Historical average models, autoregressive integrated moving average approaches, and related linear forecasting techniques provided interpretable baselines and were computationally efficient [14]. Such methods perform reasonably well when traffic patterns are stable and periodic, but their representational capacity is limited in the face of nonlinear traffic dynamics, abrupt congestion transitions, and complex inter-location dependencies.

With the growth of traffic sensing data and computational resources, machine learning methods gradually became more prominent [15]. Support vector regression, random forests, and other nonlinear predictors offered improved flexibility over traditional linear models [16]. Even so, these approaches still relied heavily on handcrafted features and often struggled to capture the coupled temporal and spatial structure of urban traffic systems in a unified manner.

Deep learning has substantially reshaped this landscape. Recurrent neural networks and their gated variants improved the modeling of sequential dependence by learning temporal patterns directly from raw traffic observations [17]. Temporal convolutional models further enhanced sequence learning through hierarchical receptive fields and efficient parallel computation [18]. More recently, attention-based architectures have demonstrated strong capacity in long-range dependency modeling and flexible representation learning, making them increasingly attractive for traffic forecasting tasks [19]. Despite these advances, the practical superiority of a particular model family often remains task-dependent, especially when prediction horizons, data quality, and structural priors differ across experimental settings.

### 2.3. Spatiotemporal Deep Learning for Traffic Prediction

A major turning point in traffic forecasting research was the recognition that temporal modeling alone is insufficient for fully characterizing urban traffic dynamics [20]. Since traffic states evolve over road networks rather than isolated locations, recent studies have increasingly focused on spatiotemporal deep learning frameworks that combine sequence modeling with explicit spatial representation.

Graph-based methods have become particularly influential in this context. By representing traffic sensors as graph nodes and their interactions as edges, these models encode the topological structure of the sensing network and capture the propagation of traffic states across space [21]. Representative architectures such as spatiotemporal graph convolutional networks and diffusion-based recurrent graph models have shown notable effectiveness in many benchmark settings [22]. Their main strength lies in the ability to integrate temporal dependency with structured spatial aggregation, thereby offering a more realistic description of multi-sensor traffic evolution.

Nevertheless, the performance of graph-based forecasting models is strongly affected by how the graph is constructed and how spatial operators are implemented. Distance-based adjacency, physical road connectivity, adaptive graph learning, and diffusion processes each impose different inductive biases, and their effectiveness can vary substantially across datasets and prediction tasks [23]. When graph construction is simplified or hyperparameter tuning is limited, the empirical advantage of graph-based models may become less pronounced.

In parallel, Transformer-based models and other attention-driven architectures have emerged as strong alternatives for traffic forecasting. These approaches do not necessarily require heavily engineered spatial operators to achieve competitive results, especially in short-horizon tasks where temporal dependency modeling plays a dominant role [24]. This observation has motivated a growing interest in reassessing the relative benefits of graph-centric and sequence-centric paradigms under unified experimental conditions.

Given these developments, recent comparative studies have increasingly emphasized methodological transparency, including consistent preprocessing, fixed training budgets, and fair evaluation protocols. Such efforts are essential for clarifying whether observed performance gains originate from genuine modeling advantages or from differences in experimental setup. In this regard, a controlled comparison across representative temporal and spatiotemporal architectures remains valuable, particularly when the goal is to understand traffic forecasting from a sensor-network perspective rather than to advocate a single model family in isolation.

### 2.4. Research Gap and Positioning

Although the existing literature has made substantial progress in short-term traffic forecasting, two interconnected gaps remain unaddressed. The first is the absence of a systematic link between sensor-level data characteristics and architecture-specific failure modes. Studies that advance a new model architecture typically report aggregate benchmark metrics but do not examine *why* a particular model fails on certain sensors or under specific data conditions.

Reproducible benchmark studies that establish these missing foundations are widely recognized as high-value contributions in adjacent machine learning domains. The ImageNet benchmark catalyzed a decade of computer vision architecture development.

The present study is positioned precisely at this gap. It develops a unified sensor-network diagnostic framework for the METR-LA dataset under the 12→3 prediction task, integrating exploratory sensor characterization, controlled benchmarking of four representative architectures (Transformer, STGCN, DCRNN, and Gated TCN), and a novel per-sensor regression analysis that quantitatively attributes model failure to identifiable data-level causes. The result is not merely a performance ranking, but an empirically grounded set of diagnostic findings and model-selection guidelines that directly motivate and constrain the design of future hybrid spatiotemporal architectures for urban traffic sensing.

Table 1 summarizes how the present study is positioned against representative research categories. Existing studies have advanced short-term traffic forecasting from classical statistical models to deep temporal networks, graph neural networks, dynamic graph learning, and multi-source sensor-aware fusion. Compared with these studies, the present work is not designed to introduce a more complex architecture, but to provide a controlled and reproducible comparison among representative forecasting paradigms. Specifically, Transformer and Gated TCN are used to examine temporal sequence modeling, while STGCN is included to evaluate road-network spatial dependency learning. Based on the METR-LA loop-detector dataset, all models are evaluated under the same 12→3 forecasting task, identical data split, fixed 50-epoch training budget, best-validation checkpoint selection, and comprehensive metrics. This design highlights the empirical contribution of this work: a fair and interpretable benchmark for understanding the relative effectiveness of temporal and graph-based models in short-horizon traffic speed prediction.

## 3. Materials and Methods

### 3.1. Dataset Description and Problem Definition

This study is conducted on the METR-LA dataset, which is widely used for short-term urban traffic forecasting. The dataset contains traffic observations collected from 207 loop detectors deployed across the Los Angeles road network, with a temporal resolution of 5 min. Although the sensing infrastructure simultaneously records speed, flow, and occupancy, traffic speed is selected as the sole prediction target in this work, since it provides a direct characterization of short-horizon traffic states and is closely associated with congestion evolution and operational efficiency. Flow and occupancy data are not used in any part of the analysis or model training.

In terms of data organization, the dataset includes both temporal and structural information. The temporal component is stored in the traffic time-series file, which records sequential observations from all sensors over the full study period. The structural component is encoded in the graph-related file, which contains the correspondence between original sensor identifiers and node indices, together with the adjacency information describing inter-sensor connectivity in the traffic network. This combination makes the dataset suitable for studying traffic forecasting as a spatiotemporal learning problem rather than an ensemble of isolated single-sensor sequences.

**Figure 1:**
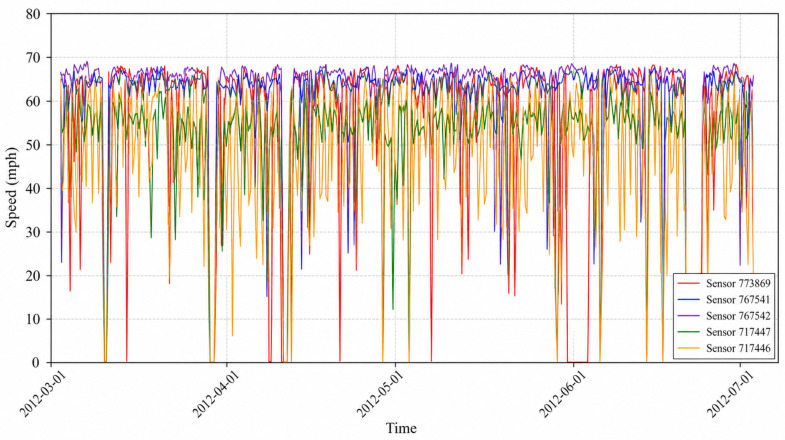
Traffic speed time series of selected sensors in the METR-LA dataset.

As shown in Figure 1, the selected sensors exhibit a relatively stable high-speed background together with recurrent short-term drops and local fluctuations. This pattern reflects the coexistence of regular traffic operation and intermittent disturbances in the sensing data. This also suggests that the forecasting task involves not only capturing persistent temporal structure but also responding to localized variations across different sensors.

**Figure 2:**
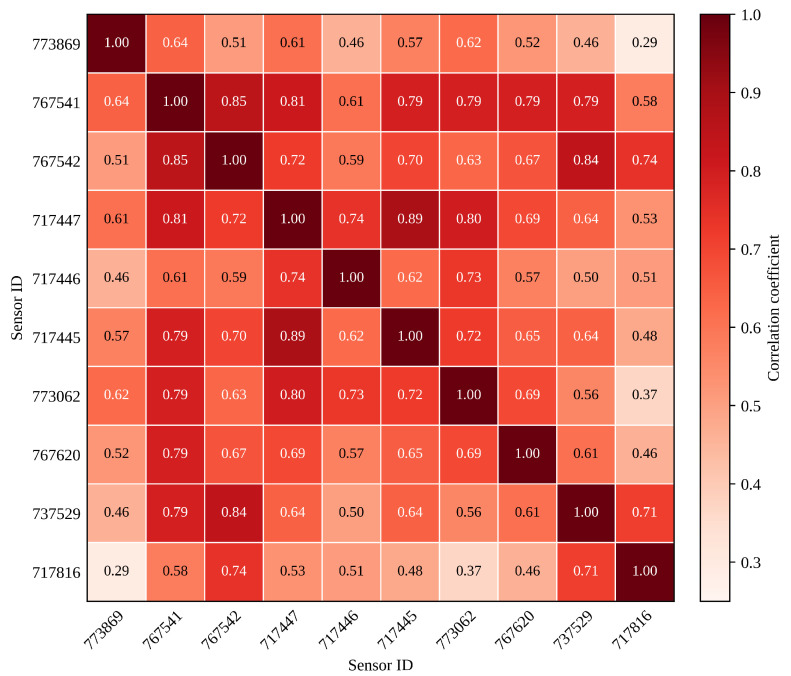
Correlation heatmap of traffic speeds among selected sensors.

Figure 2 further indicates that the sensor observations are not independent. Positive correlations are prevalent across the selected sensors, although the strength of dependence varies considerably across sensor pairs. The values shown are Pearson correlation coefficients $\rho_{ij}$ (formally defined in Section 3.3). The geographic arrangement of all 207 sensors and the graph topology derived from the distance-threshold criterion are described in Section 3.2, providing the spatial context for interpreting the correlation structure visible in Figure 2. This result supports the treatment of METR-LA as a multi-sensor spatiotemporal dataset and motivates the adoption of forecasting models that can exploit cross-sensor interactions.

The forecasting task considered in this study follows a multi-step setting. Specifically, the model takes traffic observations from the previous 12 time steps as input and predicts traffic speeds over the subsequent 3 time steps. Given the 5 min sampling interval, this corresponds to using the past 60 min to forecast traffic conditions over the next 15 min. Such a setting is practically meaningful for short-term traffic management because it captures recent traffic evolution while maintaining a prediction horizon useful for route adjustment, congestion warning, and local control support.

Let N denote the number of sensors, and let $x_{t}\;\in \;R^{N}$ represent the traffic speed vector observed at time step t. The multivariate traffic sequence over time can then be written as

$$X\;=\;\left\{x_{1},x_{2},\ldots ,x_{T}\right\},\;x_{t}\;\in \;R^{N}$$

where T denotes the total number of time steps. For each training sample, the input window is defined as

$$X^{\left(i\right)}\;=\;\left\{x_{i},x_{i+1},\ldots ,x_{i+L-1}\right\}\;\in \;R^{L\times N}$$

where L=12 is the input sequence length. The corresponding prediction target is defined as

$$Y^{\left(i\right)}\;=\;\left\{x_{i+L},x_{i+L+1},\ldots ,x_{i+L+H-1}\right\}\;\in \;R^{H\times N}$$

where H=3 is the forecasting horizon, i.e., three prediction steps corresponding to lead times of 5, 10, and 15 min ahead (denoted h=1,2,3), given the 5-min sampling interval. Accordingly, the objective of short-term traffic forecasting in this study is to learn a mapping function

$$f:R^{L\times N}\to R^{H\times N}$$

such that

$${\hat{Y}}^{\left(i\right)}\;=\;f\left(X^{\left(i\right)}\right)$$

where ${\hat{Y}}^{\left(i\right)}$ denotes the predicted traffic speeds over the next three time steps for all sensors. Under this formulation, the task is cast as a many-to-many spatiotemporal regression problem in which the model must jointly capture temporal dynamics within each sensor sequence and cross-sensor dependencies across the traffic network.

### 3.2. Data Preprocessing and Graph Construction

Because raw traffic sensor observations are often affected by data irregularities, preprocessing is required before model development. In the present study, zero-speed values are treated with particular caution during both exploratory analysis and metric design. In large-scale loop detector systems, zero readings may reflect missingness, sensor malfunction, or anomalous traffic states rather than genuine traffic standstill. Such values can distort the empirical distribution and increase the instability of percentage-based error measures. For this reason, zero-speed observations are filtered during the exploratory stage when visualizing traffic speed distributions, while the evaluation design also includes safeguards for metrics such as MAPE to reduce the influence of extremely small denominators.

In addition to zero-value handling, outlier smoothing is introduced to reduce the influence of extreme observations. Specifically, winsorization is applied using the 1st and 99th percentiles, such that overly small or overly large speed values are clipped to a more stable range. This treatment helps mitigate the adverse effect of abnormal fluctuations on model fitting while preserving the overall temporal pattern of the traffic sequence. Such a procedure is particularly useful in multisensor traffic datasets, where sporadic measurement anomalies at individual detectors may otherwise introduce instability during model training.

**Figure 3:**
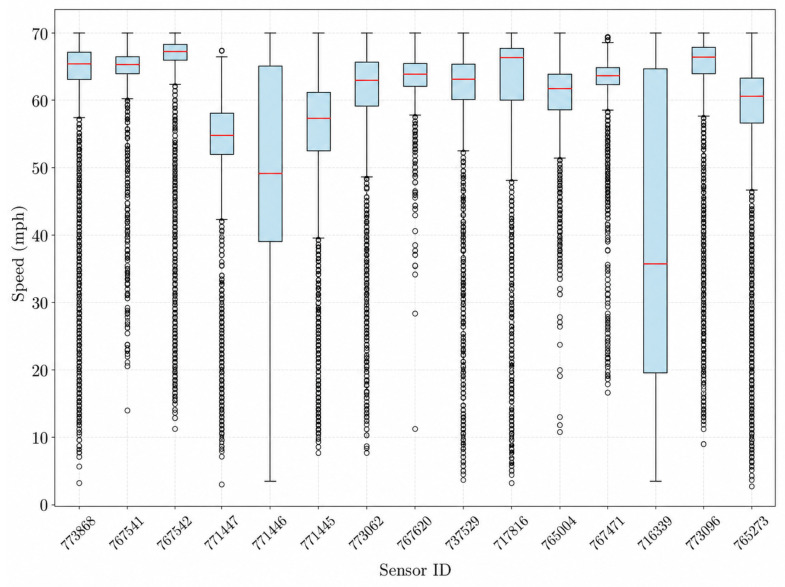
Box plots of traffic speed distributions for selected sensors. In each box plot, the lower and upper box boundaries correspond to the first quartile (Q1, 25th percentile) and the third quartile (Q3, 75th percentile), respectively; their difference is the interquartile range (IQR). The horizontal line inside the box marks the median. Whiskers extend to the most extreme data points within 1.5× IQR of the box boundaries; observations beyond the whiskers are plotted individually as outliers.

Figure 3 highlights substantial variation across sensors in terms of median speed, spread, and low-speed outliers. Some detectors maintain relatively concentrated distributions around high speeds, whereas others exhibit broader interquartile ranges and more frequent extreme values. This heterogeneity justifies the use of robust preprocessing and supports the need for sensor-wise normalization and outlier-aware treatment before model training.

To improve numerical stability and maintain comparability across sensors, the traffic speed series are standardized on a per-sensor basis. Let $x_{t}^{\left(n\right)}$ denote the observation of sensor n at time step t. For each sensor, normalization is performed using the statistics of the training set only:

$${\tilde{x}}_{t}^{\left(n\right)}\;=\;\frac{x_{t}^{\left(n\right)}\;-\;\mu_{n}}{\sigma_{n}}$$

where $\mu_{n}$ and $\sigma_{n}$ represent the mean and standard deviation of sensor n computed from the training subset. The same transformation is then applied to the validation and test subsets. This strategy avoids information leakage while ensuring that the input scale remains comparable across heterogeneous sensors.

**Figure 4:**
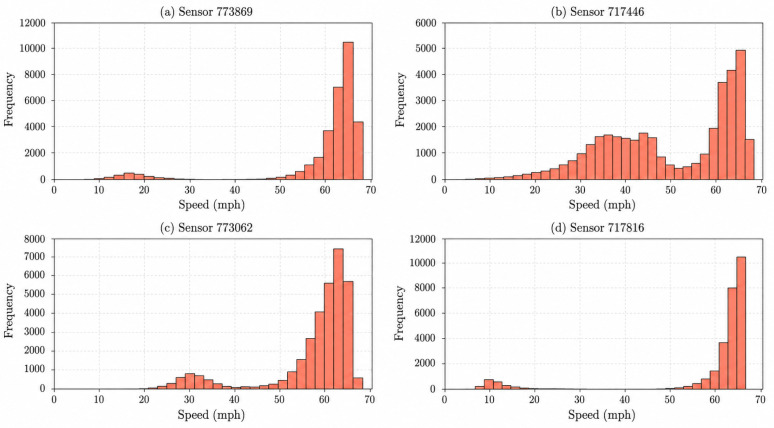
Speed histograms of selected sensors based on non-zero observations.

Figure 4 shows that the empirical speed distributions vary substantially across sensors, even after excluding zero values. Several sensors exhibit highly concentrated high-speed peaks, whereas others show wider or more mixed distributions. This observation provides additional empirical support for per-sensor standardization, since a single global scaling strategy would be less effective in accommodating such location-specific statistical differences.

Following preprocessing, the full dataset is divided into training, validation, and test subsets with a ratio of 70%/10%/20%. From the processed time series, sliding windows are constructed to generate supervised learning samples. Each input sample contains 12 consecutive historical observations across all sensors, and each target sample contains the following 3 future observations. Through this procedure, the original sequential traffic records are transformed into a sample set suitable for batch-based model training and evaluation.

To explicitly represent spatial dependency among detectors, a graph structure is constructed over the sensor network. Let G=(V,E,A) denote the traffic graph, where V is the set of sensor nodes, E is the set of edges, and $A\;\in \;R^{N\times N}$ is the adjacency matrix. In this study, the graph is built using a distance-threshold strategy, in which two sensors are connected if their physical distance satisfies a predefined neighborhood condition. This results in a binary adjacency matrix that captures local spatial proximity within the traffic sensing network. To preserve each node’s own information during graph-based aggregation, self-loops are added to the adjacency structure, yielding

$$\tilde{A}\;=\;A\;+\;I$$

where I is the identity matrix. In this study, edge weights are first computed using a Gaussian kernel applied to pre-computed road-network distances between sensor pairs:

$$W_{ij}=\exp\;\left[-{\left(\frac{d_{ij}}{\sigma_{d}}\right)}^{2}\right]$$

where $d_{ij}$ is the road-network distance between sensors *i* and *j*, and $\sigma_{d}$ is the standard deviation of all finite pairwise road-network distances in the distance matrix (note: $\sigma_{d}$ is a graph construction parameter and is distinct from the per-sensor speed standard deviation $\sigma_{n}$ defined in the preprocessing step). A binary adjacency is then obtained by thresholding at k=0.1: edges for which $W_{ij}<0.1$ are removed, and the remaining edges are set to 1. This follows the standard METR-LA graph construction protocol from the DCRNN reference implementation [5], and yields 1515 directed edges across 207 nodes (see Appendix A.1, Table A1 for full dataset parameters).

**Figure 5:**
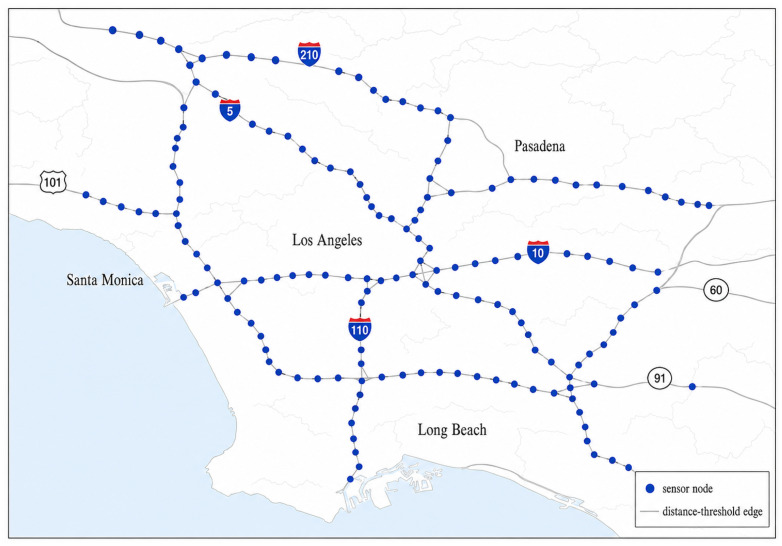
Spatial organization and Gaussian-kernel graph topology of the 207 METR-LA loop detectors. Each filled circle represents a sensor (loop detector) deployed on the Los Angeles highway network; a directed line between two circles indicates a retained graph edge. An edge is retained between sensors *i* and *j* when $W_{ij}=\exp\left[-{\left(d_{ij}/\sigma_{d}\right)}^{2}\right]\geq 0.1$, where $d_{ij}$ is the road-network distance and $\sigma_{d}$ is the standard deviation of all finite pairwise road-network distances; edges below the threshold are pruned to zero, yielding a binary adjacency matrix. The resulting graph contains 1515 directed edges. This figure provides the spatial context for interpreting the inter-sensor correlations shown in Figure 2 and the graph structure used in all spatiotemporal models.

Figure 5 confirms that the sensor deployment is spatially non-uniform: sensors cluster along major freeway corridors and interchange areas, which accounts for the block structure of high correlations observed in Figure 2. Sensors within the same corridor tend to have small road-network distances and thus high Gaussian-kernel weights $W_{ij}$, making them likely to be retained as edges, while sensors on geometrically proximate but structurally separate road segments may still fall below the k=0.1 threshold and remain disconnected. This motivates the use of road-network distance rather than Euclidean distance as the basis for graph construction.

**Figure 6:**
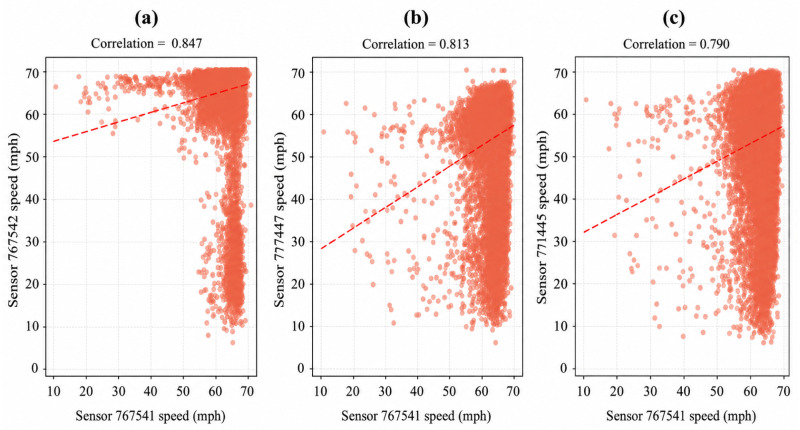
Scatter plots of strongly correlated sensor pairs. Each panel shows the joint distribution of concurrent traffic speeds for one representative sensor pair.

Figure 6 provides a pairwise view of strong cross-sensor dependence. The selected sensor pairs follow clear positive trends, indicating shared traffic dynamics, yet the point clouds remain dispersed rather than collapsing onto a single line. This suggests that the spatial relationship among sensors is substantial but not strictly deterministic, which supports the use of graph construction as a structured approximation rather than an exact physical mapping.

### 3.3. Exploratory Analysis of Traffic Sensor Data

Before model development, an exploratory analysis is conducted to characterize the statistical, temporal, and spatial properties of the traffic sensor data. This step is essential because the effectiveness of short-term forecasting depends not only on model architecture, but also on the underlying behavior of the observed traffic signals. For multi-sensor urban traffic data, descriptive analysis helps clarify whether the prediction problem is dominated by distributional skewness, temporal regularity, spatial coupling, or local irregularity, thereby providing an empirical basis for subsequent preprocessing and model selection.

Throughout this section, $x_{t}^{\left(n\right)}$ denotes the traffic speed of sensor n at time step t as defined in Section 3.2, with n=1,…,N and t=1,…,T. The per-sensor mean $\mu_{n}$ and standard deviation $\sigma_{n}$ (also defined in Section 3.2) provide the most basic description of central tendency and variability. Since traffic states can differ substantially across road segments, the distribution of $\mu_{n}$ and $\sigma_{n}$ over all sensors provides an initial indication of spatial heterogeneity in the sensing network. A relatively concentrated range of $\mu_{n}$ suggests consistent background operating conditions across detectors, whereas large variation in $\sigma_{n}$ indicates that some locations are exposed to stronger fluctuations or more frequent congestion transitions. A potential contributing factor is proximity to signalized intersections, where traffic lights induce stop-and-go cycles that amplify local speed variance; however, quantifying the correlation between $\sigma_{n}$ and distance to nearby traffic signals would require additional infrastructure data (e.g., signal controller locations) that are not available in the METR-LA dataset, and is therefore beyond the scope of the present study.

To further characterize the empirical distribution of traffic speed, quantile-based statistics are also considered. For each sensor n, let $Q_{p}^{\left(n\right)}$ denote the empirical p-th quantile of the sequence ${\left\{x_{t}^{\left(n\right)}\right\}}_{t=1}^{T}$. In particular, the interquartile range (IQR) is defined as

$${\mathrm{IQR}}_{n}\;=\;Q_{0.75}^{\left(n\right)}\;-\;Q_{0.25}^{\left(n\right)}$$

Compared with variance-based measures, the interquartile range is more robust to extreme values and is therefore useful for assessing the spread of traffic speed in the presence of sensor anomalies. The IQR is directly visible in the box plots of Figure 3, where the box height equals ${\mathrm{IQR}}_{n}$ for each displayed sensor. If a sensor exhibits both a large $\sigma_{n}$ and a large ${\mathrm{IQR}}_{n}$, its variability is likely driven by genuine traffic regime changes. By contrast, a disproportionately large $\sigma_{n}$ relative to ${\mathrm{IQR}}_{n}$ may suggest the presence of abnormal readings or isolated outliers.

From a distributional perspective, the traffic speed data exhibit a clear concentration around a moderate-to-high speed range, together with an extended lower tail. This pattern is consistent with the operational characteristics of urban freeway traffic, where uncongested conditions occupy a substantial portion of the observation period, while congestion episodes produce lower-speed deviations. Such a distribution implies that the forecasting task is not fully symmetric: the model must perform well not only under common, relatively stable states, but also under compressed low-speed regimes that may be less frequent yet operationally more critical. The existence of this left-skewed behavior also justifies the use of robust preprocessing and multiple error metrics in later evaluation.

Zero-value behavior deserves separate examination because it carries both statistical and operational implications. Let the zero-value indicator of sensor n at time t be defined as

$$z_{t}^{\left(n\right)}\;=\;\left\{\begin{array}{cc} 1, & x_{t}^{\left(n\right)}\;=\;0\\ 0, & x_{t}^{\left(n\right)}\;>\;0 \end{array}\right.\;$$

The zero-value ratio for sensor n can then be written as

$$r_{n}^{\left(0\right)}\;=\;\frac{1}{T}\sum_{t=1}^{T}\;z_{t}^{\left(n\right)}$$

At the network level, the aggregate zero-value ratio is

$$r^{\left(0\right)}\;=\;\frac{1}{\mathrm{NT}}\sum_{n=1}^{N}\;\sum_{t=1}^{T}\;z_{t}^{\left(n\right)}$$

This quantity reflects the global prevalence of zero readings across the dataset. In practical traffic sensing systems, zero observations may arise from actual standstill, detector malfunction, communication loss, or missing-value encoding. Consequently, their interpretation should remain cautious. A moderate but non-negligible $r^{\left(0\right)}$ indicates that zero readings are systematic enough to influence distribution analysis and metric computation, especially for percentage-based measures. This observation further supports the preprocessing strategy introduced earlier.

Temporal variability is another important aspect of exploratory analysis. For each sensor n, the temporal increment over one step can be defined as

$$\Delta x_{t}^{\left(n\right)}\;=\;x_{t}^{\left(n\right)}\;-\;x_{t-1}^{\left(n\right)},\;t\;=\;2,\;\ldots ,\;T$$

The magnitude of $\Delta x_{t}^{\left(n\right)}$ characterizes short-term local fluctuation, while its empirical distribution reflects the smoothness or volatility of the traffic process. When most increments remain close to zero, the sequence evolves gradually and is likely to be more predictable over short horizons. Conversely, large and irregular increments indicate abrupt state transitions that may increase forecasting difficulty. In the present dataset, representative sensor trajectories show recognizable daily cycles together with localized fluctuations, suggesting that both periodic structure and transient deviations coexist in the traffic process.

To quantify temporal regularity at a broader scale, the average traffic profile over fixed intraday positions can be introduced. Since the observations are recorded every 5 min, each day contains M=288 time steps. Let d index the day and m index the position within the day. For sensor n, the average intraday speed profile is defined as

$${\bar{x}}_{m}^{\left(n\right)}\;=\;\frac{1}{D}\sum_{d=1}^{D}\;x_{d,m}^{\left(n\right)}$$

where D is the number of days in the dataset and $x_{d,m}^{\left(n\right)}$ denotes the observation at intraday step m on day d. This representation makes it possible to examine recurrent diurnal patterns, including morning and evening peaks, midday stabilization, and late-night recovery. The presence of structured intraday cycles indicates that short-term traffic forecasting is not purely reactive; it also benefits from regular temporal context embedded in the sensing data.

A related issue concerns variation across different day types. Let $D_{w}$ and $D_{e}$ denote the sets of weekday and weekend indices, respectively. The corresponding average intraday profiles can be written as

$${\bar{x}}_{m,w}^{\left(n\right)}\;=\;\frac{1}{\left|D_{w}\right|}\sum_{d\in D_{w}}\;x_{d,m}^{\left(n\right)}$$

and

$${\bar{x}}_{m,e}^{\left(n\right)}\;=\;\frac{1}{\left|D_{e}\right|}\sum_{d\in D_{e}}\;x_{d,m}^{\left(n\right)}$$

The difference

$$\delta_{m}^{\left(n\right)}\;=\;{\bar{x}}_{m,w}^{\left(n\right)}\;-\;{\bar{x}}_{m,e}^{\left(n\right)}$$

measures the systematic contrast between weekday and weekend traffic behavior at each time position. If $\delta_{m}^{\left(n\right)}$ is consistently nonzero over extended intervals, then weekly periodicity forms an additional source of predictable structure. In practice, this distinction often reflects commuting demand, work-related trip concentration, and more regular peak periods on weekdays than on weekends.

Beyond temporal analysis, spatial correlation across sensors is a defining property of the dataset. For any pair of sensors i and j, the Pearson correlation coefficient between their speed sequences—visualized in Figure 2—is given by

$$\rho_{\mathrm{ij}}\;=\;\frac{\sum_{t=1}^{T}\;\;\left(x_{t}^{\left(i\right)}\;-\;\mu_{i}\right)\left(x_{t}^{\left(j\right)}\;-\;\mu_{j}\right)}{\sqrt{\sum_{t=1}^{T}\;\;{\left(x_{t}^{\left(i\right)}\;-\;\mu_{i}\right)}^{2}}\sqrt{\sum_{t=1}^{T}\;\;{\left(x_{t}^{\left(j\right)}\;-\;\mu_{j}\right)}^{2}}}$$

Stacking all pairwise coefficients yields the correlation matrix

$$C=\left[\rho_{\mathrm{ij}}\right]\in R^{N\times N}$$

This matrix provides a descriptive view of cross-sensor dependency independent of any preimposed graph structure. When blocks of high correlation appear in C, they suggest the existence of spatially coherent traffic regions or corridor-level interactions. Such clusters provide empirical support for the use of graph-based or multivariate forecasting models, since independent per-sensor prediction would ignore meaningful shared dynamics.

To summarize the overall strength of spatial dependence derived from C, a sensor-level average correlation measure may be introduced as

$${\bar{\rho }}_{i}\;=\;\frac{1}{N-1}\sum_{\begin{array}{c} j=1\\ j\neq i \end{array}}^{N}\;\rho_{\mathrm{ij}}$$

A relatively high ${\bar{\rho }}_{i}$ indicates that sensor i evolves in close coordination with the rest of the network, whereas a low value may reflect localized or isolated dynamics. From a modeling perspective, this distinction is important because highly coupled sensors are more likely to benefit from explicit spatial aggregation, while weakly coupled sensors may rely more on local temporal history.

The combined exploratory findings suggest that the METR-LA traffic data exhibit three salient characteristics. At the distributional level, traffic speed is concentrated within a moderate operating range but retains a pronounced lower tail associated with congestion states. At the temporal level, the data contain both regular intraday structure and short-term local fluctuations, implying that successful forecasting requires a balance between pattern extraction and dynamic responsiveness. At the spatial level, inter-sensor correlation is evident and often structured, which supports the use of models capable of exploiting cross-location dependency. Taken together, these properties motivate the subsequent comparative evaluation of temporal and spatiotemporal forecasting architectures under a unified experimental setting.

### 3.4. Forecasting Models

To ensure a transparent comparison across different forecasting paradigms, all models in this study are formulated under a unified prediction framework. For each sample, the input is a multivariate traffic sequence

$$X\;\in \;R^{L\times N}$$

where L=12 denotes the historical sequence length and N denotes the number of sensors. The output is the corresponding multi-step forecast

$$\hat{Y}\;\in \;R^{H\times N}$$

where H=3 is the prediction horizon. Under this setting, the learning objective is to estimate a nonlinear mapping

$$\hat{Y}\;=\;f(X;\Theta )$$

where f(·) denotes the forecasting model parameterized by Θ. Although all four models share the same input-output structure, they differ substantially in how temporal dependency, spatial correlation, and cross-sensor interaction are represented. This difference is central to the comparative analysis carried out in the following sections.

Within the present framework, the Transformer is adopted as a sequence-oriented baseline with strong temporal representation capacity. Given the input sequence X, the model first projects the original observations into a latent feature space and then applies self-attention to capture dependency patterns across time steps. Let Q,K, and V denote the query, key, and value matrices generated from the input representation. The self-attention operation can be written as

$$\mathrm{Attention}(Q,\;K,\;V)\;=\;\mathrm{softmax}\left(\frac{QK^{⊤}}{\sqrt{d_{k}}}\right)V$$

where $d_{k}$ is the feature dimension used for scaling. This mechanism allows the model to adaptively assign importance weights to different historical positions when constructing future predictions. Compared with recurrent architectures, the Transformer is more effective in modeling long-range temporal interactions and benefits from parallel computation. In the context of short-term traffic forecasting, such a design is especially suitable when temporal regularity and cross-time dependency dominate the predictive structure, even if the spatial relationship is not explicitly encoded through graph operators.

The STGCN model is introduced to capture spatial and temporal dependencies in an integrated manner. It combines graph convolution for spatial aggregation with temporal convolution for sequential feature extraction. Let A denote the adjacency matrix of the traffic graph and let $\tilde{A}\;=\;A\;+\;I$ be the adjacency matrix with self-loops. The normalized graph propagation can be expressed as

$$\hat{A}\;=\;D^{\;-\;\frac{1}{2}}\tilde{A}D^{\;-\;\frac{1}{2}}$$

where D is the degree matrix associated with $\tilde{A}$. For a graph feature matrix Z, the graph convolution operation takes the general form

$$Z^{\prime }\;=\;\hat{A}\mathrm{ZW}$$

where W is a learnable weight matrix. This operation enables each sensor node to aggregate information from its local neighbors in the graph. In parallel, temporal convolution is applied along the time dimension to model sequential variation. Through the alternating combination of temporal and graph operations, STGCN learns short-term traffic evolution under explicit spatial constraints. Such a structure is well suited to traffic sensing networks in which local road proximity is expected to influence speed propagation.

The DCRNN model extends graph-based traffic forecasting by introducing recurrent sequence modeling together with diffusion-style spatial propagation. Rather than relying on ordinary graph convolution alone, DCRNN interprets traffic flow dependency as a directed diffusion process on the graph, to better reflect the asymmetric propagation of traffic states. Let $h_{t}$ denote the hidden state at time step t. The recurrent update can be represented in generic form as

$$h_{t}\;=\;\Phi \left(x_{t},\;h_{t-1};\;\Theta \right)$$

where Φ(·) denotes a gated recurrent transition equipped with graph diffusion operators. The predicted sequence is then generated through recursive hidden-state evolution over time. Compared with purely convolutional spatiotemporal models, DCRNN is designed to strengthen the representation of sequential state transition while maintaining awareness of graph-based spatial influence. This architecture is particularly appropriate when the traffic process exhibits both directional propagation and strong temporal memory. At the same time, its empirical effectiveness depends considerably on the quality of the graph structure and the adequacy of diffusion parameterization.

The Gated TCN serves as a temporal convolutional alternative that emphasizes hierarchical sequence modeling through dilated causal convolutions. For a one-dimensional temporal signal $x_{t}$, the dilated convolution at time t can be written as

$$y_{t}\;=\;\sum_{k=0}^{K-1}\;w_{k}x_{t-\mathrm{dk}}$$

where K is the kernel size, d is the dilation factor, and $w_{k}$ denotes the convolution weight. By increasing the dilation factor across layers, the model expands its temporal receptive field without requiring excessively deep recursion. To enhance nonlinear filtering and dynamic selection of useful temporal features, a gating mechanism is incorporated into the convolutional block. A typical gated output can be written as

$$Z\;=\;\tan h\left(W_{f}*X\right)⊙\sigma \left(W_{g}*X\right)$$

where $W_{f}$ and $W_{g}$ are convolution kernels, σ(·) is the sigmoid activation, and ⊙ denotes elementwise multiplication. This formulation enables the model to regulate the contribution of latent temporal features adaptively. In traffic forecasting, Gated TCN is advantageous when short- and medium-range temporal patterns are informative and efficient parallel sequence processing is desired. However, because it lacks an inherent spatial modeling component, its effectiveness in multi-sensor forecasting depends on whether temporal representations alone are sufficient to compensate for the absence of graph interaction mechanisms.

Taken together, these four models represent distinct yet complementary forecasting paradigms. The Transformer emphasizes adaptive temporal dependency learning through attention; STGCN combines local graph aggregation with temporal convolution; DCRNN integrates recurrent state transition with diffusion-based spatial propagation; and Gated TCN focuses on efficient hierarchical temporal modeling through gated dilated convolutions. Evaluating them under the same data preprocessing, graph construction, and forecasting horizon makes it possible to compare the relative contributions of temporal modeling strength and explicit spatial representation in short-term traffic forecasting.

Motivated by the comparative findings, this study further proposes a lightweight hybrid architecture, GETFormer, which combines the temporal modeling strength of the Transformer with a one-hop graph convolution layer to explicitly inject local sensor-network information. The design is intentionally minimal: rather than replacing the Transformer backbone with a complex spatiotemporal encoder, GETFormer augments it with a gated residual fusion module that adaptively controls the contribution of graph-enhanced features, preventing noisy or weakly coupled neighbors from degrading the temporal representation.

Given the input $X\in R^{B\times L\times N\times 1}$, GETFormer first maps observations to a latent dimension *d* via a shared linear projection, adds positional and learnable node embeddings, and then applies a Transformer encoder independently across the time dimension of each sensor. The last time-step hidden state $H_{T}\in R^{B\times N\times d}$ serves as the per-sensor temporal representation. A single-hop graph convolution then computes the spatially aggregated representation:

(1) $$H_{G}=\hat{A}\;H_{T}\;W_{G}$$

where $\hat{A}=D^{-\frac{1}{2}}\tilde{A}D^{-\frac{1}{2}}$ is the symmetrically normalized adjacency matrix with self-loops and $W_{G}$ is a learnable weight matrix. To avoid unconditional propagation of spatial noise, a gated residual fusion module combines $H_{T}$ and $H_{G}$ through an adaptive gate:

(2) $$G=\sigma \;\left(\mathrm{MLP}\;\left([H_{T};\;H_{G}]\right)\right),\;H=\mathrm{LayerNorm}\;\left(H_{T}+G⊙H_{G}\right)$$

where σ(·) is the sigmoid function and ⊙ denotes element-wise multiplication. Finally, a two-layer MLP prediction head maps H to the *H*-step forecast $\hat{Y}\in R^{B\times H\times N}$. GETFormer is trained under the same 50-epoch budget, Adam optimizer, learning rate, batch size, and checkpoint selection rule as the four baseline models, ensuring a fair comparison. The additional parameter count introduced by the graph convolution and gated fusion module is modest relative to the Transformer encoder, keeping the architecture lightweight.

### 3.5. Training Strategy and Evaluation Metrics

To ensure that the comparative analysis is both fair and reproducible, all forecasting models are trained and evaluated under a unified experimental protocol. This design minimizes the influence of inconsistent optimization settings and makes the observed performance differences more directly attributable to the modeling frameworks themselves. In particular, the same data partitions, prediction horizon, preprocessing pipeline, and evaluation criteria are applied to all models throughout the experiment.

A fixed training budget is adopted for all models. Let $\Theta^{\left(e\right)}$ denote the model parameters after the e th training epoch. Each model is trained for a total of E=50 epochs, yielding the parameter trajectory

$$\Theta^{\left(1\right)},\;\Theta^{\left(2\right)},\;\ldots ,\;\Theta^{\left(E\right)}$$

Using an identical epoch budget ensures no model benefits disproportionately from a longer training process. This setting is particularly important in comparative forecasting studies, since unequal optimization effort may otherwise obscure the intrinsic differences between architectures.

Model selection is based on a unified validation criterion. Let $L_{\mathrm{val}\;}^{\left(e\right)}$ denote the validation error measured after epoch e. The optimal checkpoint is selected according to

$$e^{*}\;=\;\arg\min_{1\leq e\leq E}\;L_{\mathrm{val}}^{\left(e\right)}$$

The final test evaluation is then conducted using the restored parameter set $\Theta^{\left(e^{*}\right)}$ rather than the last-epoch model. This strategy prevents overfitting in later training stages from distorting the final comparison and ensures that each model is assessed at its best validation state under the same training budget.

Another important consideration concerns the physical meaning of validation. In this study, validation is conducted in the original traffic speed unit, namely miles per hour (mph), rather than only in normalized feature space. Suppose that $\tilde{y}$ and $\hat{\tilde{y}}$ denote the normalized ground truth and prediction, respectively. The inverse transformation for sensor n is given by

$$y^{\left(n\right)}\;=\;\sigma_{n}{\tilde{y}}^{\left(n\right)}\;+\;\mu_{n},\;{\hat{y}}^{\left(n\right)}\;=\;\sigma_{n}{\hat{\tilde{y}}}^{\left(n\right)}\;+\;\mu_{n}$$

where $\mu_{n}$ and $\sigma_{n}$ are the mean and standard deviation computed from the training subset. By evaluating model performance after inverse transformation, the validation results remain directly interpretable in real traffic units. This design is more appropriate for practical forecasting tasks because it aligns model selection with physically meaningful prediction accuracy.

To maintain computational stability during validation and testing, mini-batch evaluation is used instead of processing the entire validation or test set in a single forward pass. Let $B_{1},\;B_{2},\;\ldots ,\;B_{M}$ denote the batches generated from the evaluation set, where M is the total number of batches. For any error metric l(·) defined at the batch level, the overall evaluation result can be written as

$$L_{\mathrm{eval}\;}\;=\;\frac{1}{\sum_{m=1}^{M}\;\;\left|B_{m}\right|}\sum_{m=1}^{M}\;\left|B_{m}\right|l\left(B_{m}\right)$$

where $\left|B_{m}\right|$ denotes the number of samples in batch $B_{m}$. This formulation improves memory efficiency while preserving the consistency of full-set evaluation.

To comprehensively assess forecasting performance, multiple metrics are employed. Let $y_{t}^{\left(n\right)}$ and ${\hat{y}}_{t}^{\left(n\right)}$ denote the true and predicted traffic speeds, respectively, for sensor n at forecasted time step t. For convenience, suppose that the total number of evaluated prediction points is S, after flattening across all samples, forecast horizons, and sensors.

The mean absolute error (MAE) is defined as

$$\mathrm{MAE}\;=\;\frac{1}{S}\sum_{s=1}^{S}\;\left|y_{s}\;-\;{\hat{y}}_{s}\right|$$

MAE provides a direct measure of average absolute deviation in physical units and is easily interpretable for short-term traffic forecasting.

The root mean squared error (RMSE) is given by

$$\mathrm{RMSE}\;=\;\sqrt{\frac{1}{S}\sum_{s=1}^{S}\;\;{\left(y_{s}\;-\;{\hat{y}}_{s}\right)}^{2}}$$

Compared with MAE, RMSE penalizes larger deviations more heavily and is therefore more sensitive to occasional large forecasting errors.

To assess goodness of fit relative to the variance of the observed data, the coefficient of determination $R^{2}$ is also reported:

$$R^{2}\;=\;1-\frac{\sum_{s=1}^{S}\;\;{\left(y_{s}\;-\;{\hat{y}}_{s}\right)}^{2}}{\sum_{s=1}^{S}\;\;{\left(y_{s}\;-\;\bar{y}\right)}^{2}}$$

where

$$\bar{y}\;=\;\frac{1}{S}\sum_{s=1}^{S}\;y_{s}$$

is the mean of the true observations. A higher $R^{2}$ indicates that the model explains a larger proportion of the variation in the observed traffic speeds.

Percentage-based metrics are included to evaluate relative error behavior. The mean absolute percentage error (MAPE) is defined as

$$\mathrm{MAPE}\;=\;\frac{100\%}{S}\sum_{s=1}^{S}\;\left|\frac{y_{s}\;-\;{\hat{y}}_{s}}{y_{s}}\right|$$

However, since traffic speed data may contain very small values, MAPE can become unstable when the denominator approaches zero. To reduce this effect, the computation is guarded by excluding or thresholding extremely small speed values during evaluation, thereby preventing disproportionate inflation of the metric.

The symmetric mean absolute percentage error (SMAPE) is given by

$$\mathrm{SMAPE}\;=\;\frac{100\%}{S}\sum_{s=1}^{S}\;\frac{2\left|y_{s}\;-\;{\hat{y}}_{s}\right|}{\left|y_{s}\right|\;+\;\left|{\hat{y}}_{s}\right|}$$

Unlike MAPE, SMAPE normalizes the absolute error using both the true and predicted values, which generally leads to greater numerical stability when the observed traffic speed is low.

The weighted absolute percentage error (WAPE) is defined as

$$\mathrm{WAPE}\;=\;100\%\times \frac{\sum_{s=1}^{S}\;\;\left|y_{s}\;-\;{\hat{y}}_{s}\right|}{\sum_{s=1}^{S}\;\;\left|y_{s}\right|}$$

WAPE measures the total absolute error relative to the total magnitude of the observed signal and is less sensitive to individual near-zero observations than MAPE.

Taken together, these metrics provide complementary views of forecasting quality. MAE and RMSE quantify absolute prediction deviation in physically meaningful units, $R^{2}$ evaluates explanatory strength relative to signal variance, and MAPE, SMAPE, and WAPE characterize relative error behavior from different perspectives. Under the unified training and validation strategy described above, this metric set enables a more balanced assessment of forecasting models than any single indicator alone.

With the dataset, preprocessing strategy, exploratory analysis, model designs, and evaluation protocol now specified, the following section presents the comparative experimental results for the four forecasting architectures under the 12→3 traffic prediction setting.

## 4. Results

### 4.1. Overall Quantitative Comparison

The overall performance of all five models—four baselines and the proposed GETFormer—was evaluated under the unified 12→3 prediction setting using MAE, RMSE, R^2^, MAPE, SMAPE, and WAPE. All models were trained and assessed under the same preprocessing pipeline, data partition, epoch budget, and checkpoint selection rule.

Table 2 confirms a clear performance hierarchy. GETFormer achieves the best results across all six evaluation metrics, attaining an MAE of 3.34 mph, RMSE of 7.86, R^2^ of 0.882, MAPE of 7.41%, SMAPE of 29.74%, and WAPE of 6.58%. Compared with the Transformer baseline, GETFormer reduces MAE by 0.18 mph (5.11% relative reduction), RMSE by 0.37, and WAPE by 0.36 percentage points, while increasing R^2^ from 0.870 to 0.882. These consistent improvements across both absolute and relative metrics confirm that the one-hop graph convolution with gated residual fusion provides complementary spatial cues that the Transformer alone cannot capture.

Among the four baselines, the Transformer remains the strongest, followed by STGCN, Gated TCN, and DCRNN. STGCN benefits from explicit graph-based spatial aggregation but is constrained by the simplified distance-threshold graph construction. DCRNN yields the weakest results: its multi-step diffusion-recurrent formulation is susceptible to zero-value contamination in the sensor network, as quantified in Section 4.5. Gated TCN outperforms DCRNN on all metrics but lacks any spatial modeling, confirming that temporal convolution alone is insufficient for competitive multi-sensor forecasting in this setting.

Taken together, the results show that temporal representation remains the dominant factor at the 15-min horizon, but GETFormer demonstrates that a lightweight one-hop spatial enhancement, when controlled by an adaptive gate, provides a consistent and measurable improvement over a strong temporal baseline.

### 4.2. Error Distribution Analysis

Aggregate metrics provide an overall assessment of forecasting quality, but they do not fully reveal how prediction errors are distributed. To further examine model behavior, the residual patterns of all five models are analyzed in this subsection. This perspective is useful for identifying differences in error concentration, dispersion, and stability across forecasting architectures.

**Figure 7:**
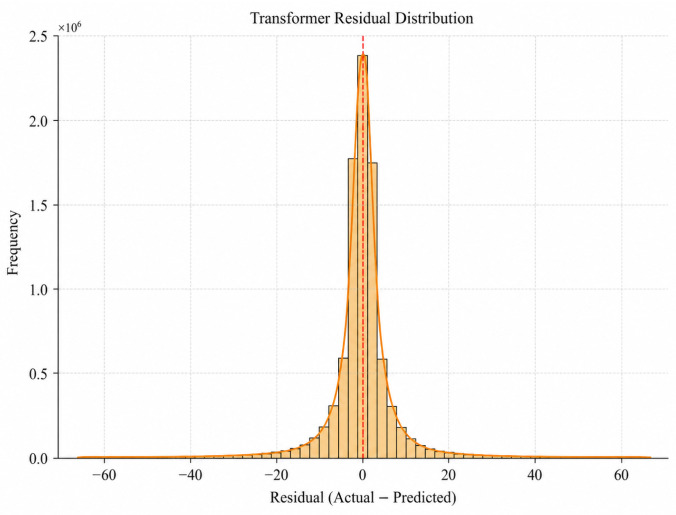
Residual distribution of Transformer. The solid curve is the kernel density estimate (KDE) of prediction residuals; the dashed curve is a fitted normal distribution shown as a reference.

**Figure 8:**
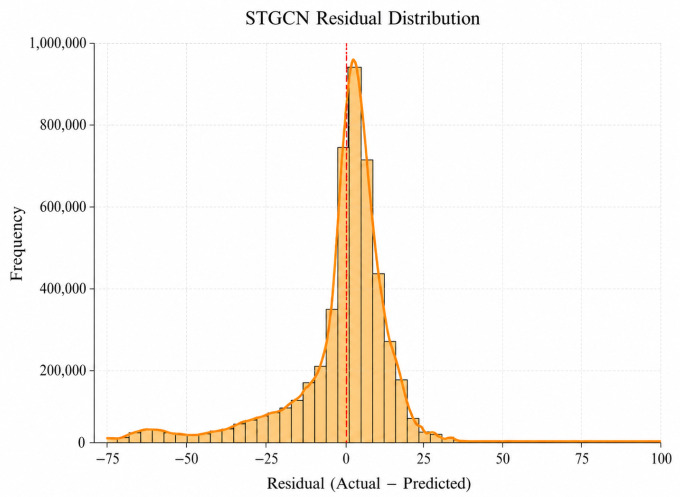
Residual distribution of STGCN. The solid curve is the kernel density estimate (KDE) of prediction residuals; the dashed curve is a fitted normal distribution shown as a reference.

**Figure 9:**
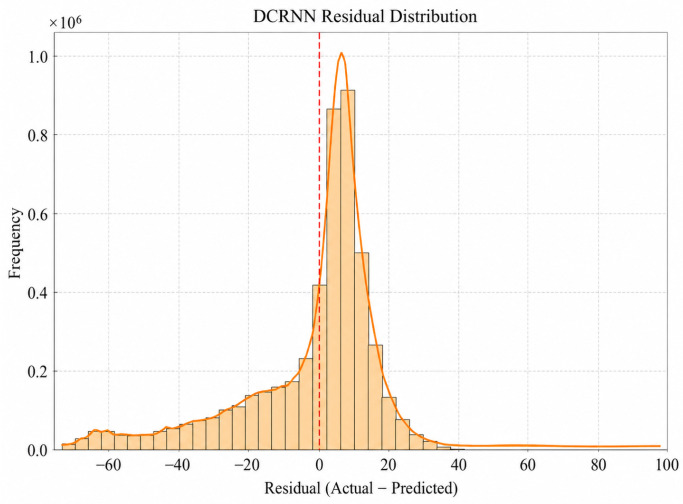
Residual distribution of DCRNN. The solid curve is the kernel density estimate (KDE) of prediction residuals; the dashed curve is a fitted normal distribution shown as a reference.

**Figure 10:**
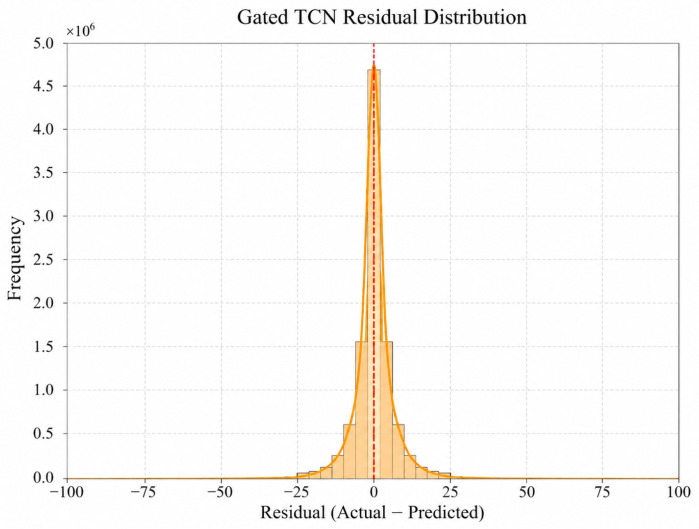
Residual distribution of Gated TCN. The solid curve is the kernel density estimate (KDE) of prediction residuals; the dashed curve is a fitted normal distribution shown as a reference. The light-yellow shaded area represents the histogram of raw residual counts (background).

**Figure 11:**
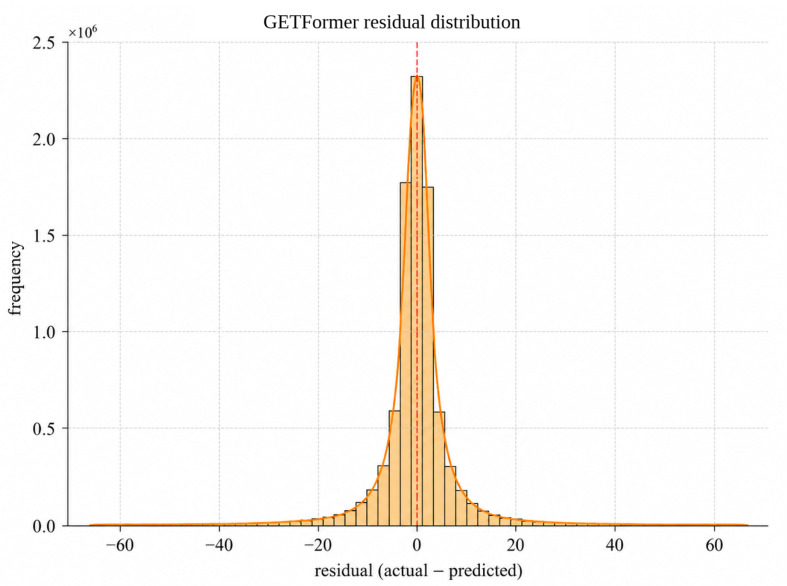
Residual distribution of GETFormer. The solid curve is the kernel density estimate (KDE) of prediction residuals; the dashed curve is a fitted normal distribution shown as a reference.

Figure 7, Figure 8, Figure 9, Figure 10 and Figure 11 provide a detailed view of the error structures underlying the aggregate metrics. GETFormer (Figure 11) exhibits the most concentrated residual distribution, with a tighter peak around zero and shorter tails compared with the Transformer (Figure 7), indicating lower bias and greater prediction stability. STGCN (Figure 8) shows a broader distribution; its forecasts remain generally reasonable but are less tightly centered. The residuals of DCRNN (Figure 9) are more dispersed with a heavier tail, reflecting systematic instability caused by zero-value contamination in the diffusion encoder. Gated TCN (Figure 10) shows the weakest residual behavior among the four baselines, with pronounced dispersion and visible extreme deviations. GETFormer’s tighter residual profile confirms that the gated graph enhancement reduces prediction variance without increasing bias, offering a more stable error distribution than any of the compared baselines.

### 4.3. Prediction-Observation Consistency Analysis

Beyond error magnitude, it is instructive to examine how closely the predicted values align with the observed traffic speeds. Prediction-observation consistency reflects whether a model can preserve a credible mapping between historical traffic states and future targets over the dominant speed range.

**Figure 12:**
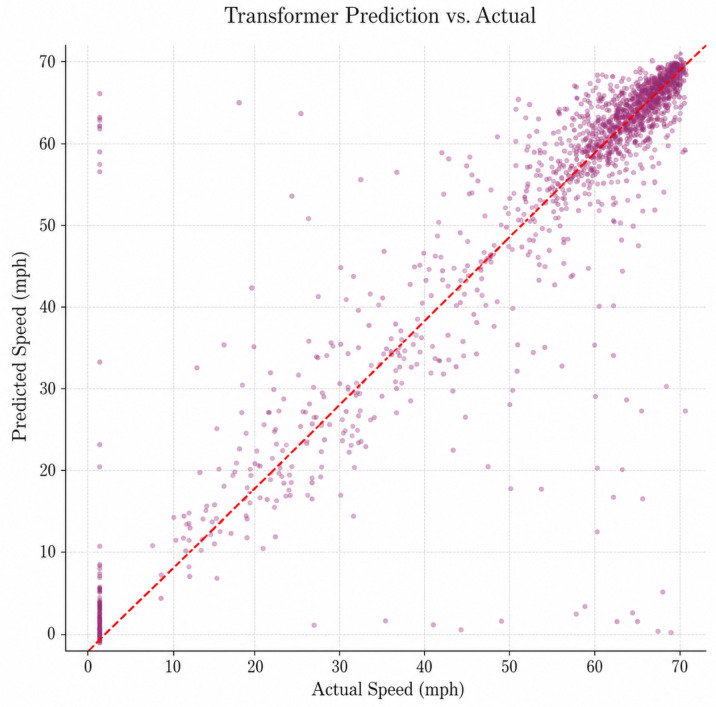
Prediction versus actual values for Transformer. Each point represents one sensor–timestep prediction. The dashed diagonal line is the perfect-prediction reference ($\hat{y}=y$).

**Figure 13:**
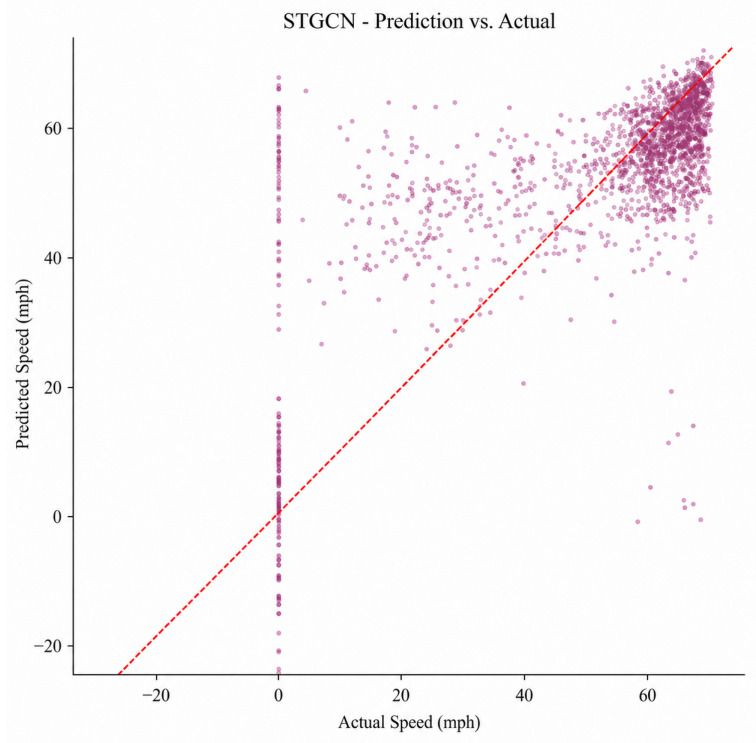
Prediction versus actual values for STGCN. Each point represents one sensor–timestep prediction. The dashed diagonal line is the perfect-prediction reference ($\hat{y}=y$).

**Figure 14:**
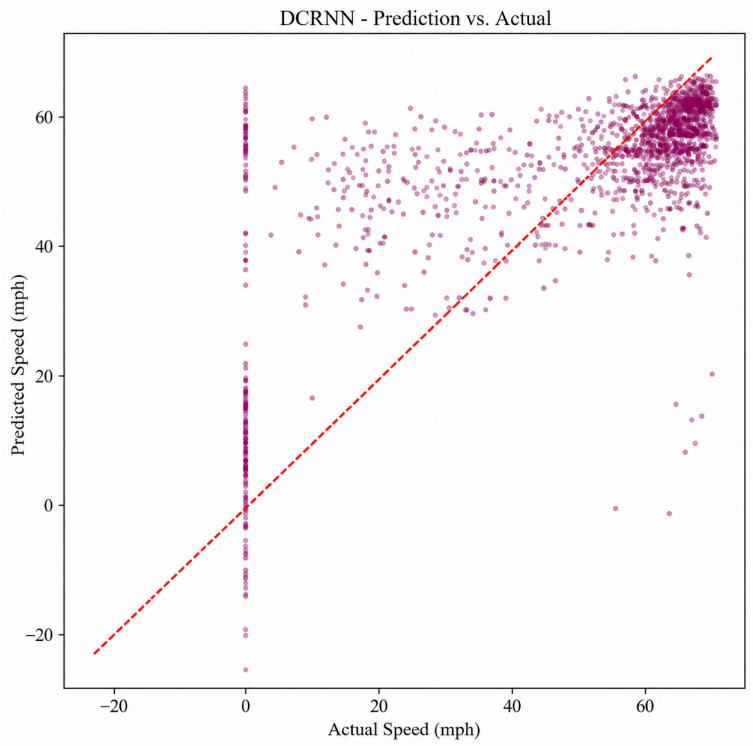
Prediction versus actual values for DCRNN. Each point represents one sensor–timestep prediction. The dashed diagonal line is the perfect-prediction reference ($\hat{y}=y$).

**Figure 15:**
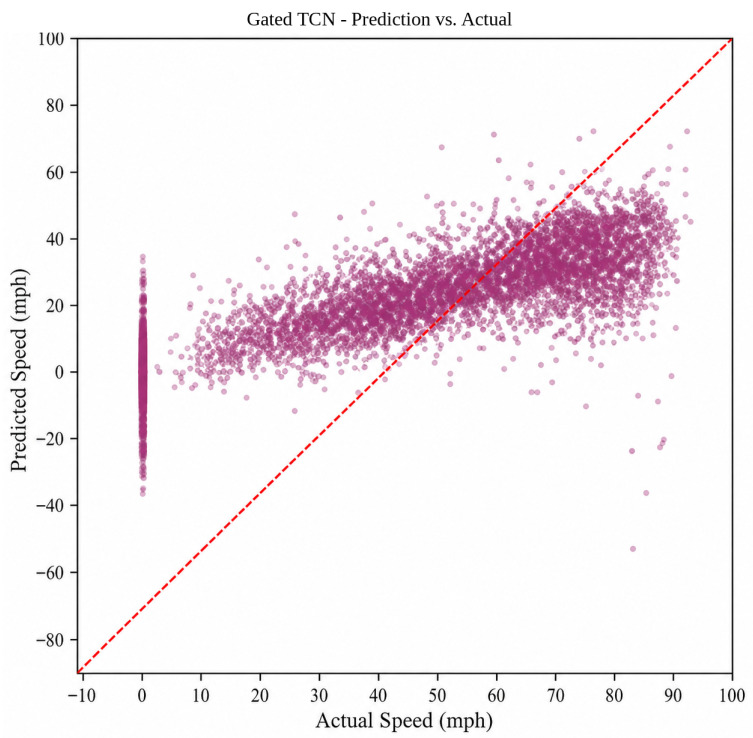
Prediction versus actual values for Gated TCN. Each point represents one sensor–timestep prediction. The dashed diagonal line is the perfect-prediction reference ($\hat{y}=y$).

**Figure 16:**
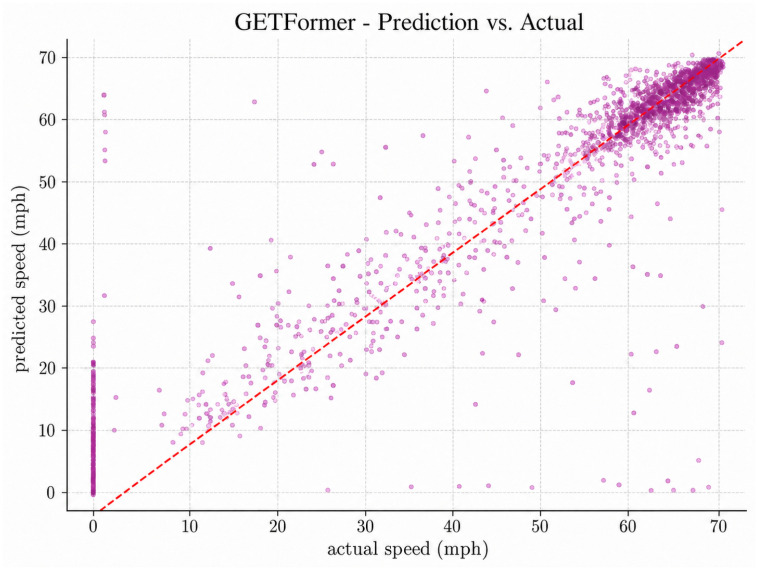
Prediction versus actual values for GETFormer. Each point represents one sensor–timestep prediction. The dashed diagonal line is the perfect-prediction reference ($\hat{y}=y$).

As shown in Figure 12, Figure 13, Figure 14, Figure 15 and Figure 16, GETFormer (Figure 16) produces the tightest alignment around the ideal diagonal across the full speed range, indicating the strongest pointwise agreement between predicted and observed traffic speeds. Compared with the Transformer (Figure 12), GETFormer’s scatter is marginally less diffuse, particularly in the moderate-speed region where spatial coupling among neighboring sensors is most informative. STGCN (Figure 13) also captures the main trend but with more spread. DCRNN (Figure 14) shows a compressed prediction pattern with outputs concentrated in a narrow band, suggesting limited responsiveness to the full speed range. Gated TCN (Figure 15) has the least regular scatter, with pronounced outlying predictions reflecting insufficient calibration. A forecasting model should not only minimize average error but also preserve a stable prediction-observation relationship; the consistency perspective confirms that GETFormer’s graph-enhanced fusion improves structural alignment over the Transformer baseline.

### 4.4. Local Temporal Tracking Analysis

Short-term traffic forecasting is inherently dynamic, and model performance should therefore also be assessed from the perspective of local temporal tracking. While global metrics summarize average behavior, they do not fully capture how well a model follows short-term fluctuations, sudden drops, or recovery patterns in traffic speed.

**Figure 17:**
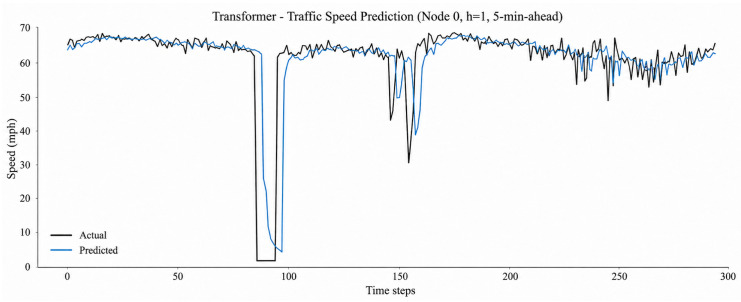
Local temporal prediction of Transformer (Node 0, 5-min-ahead horizon, h=1).

**Figure 18:**
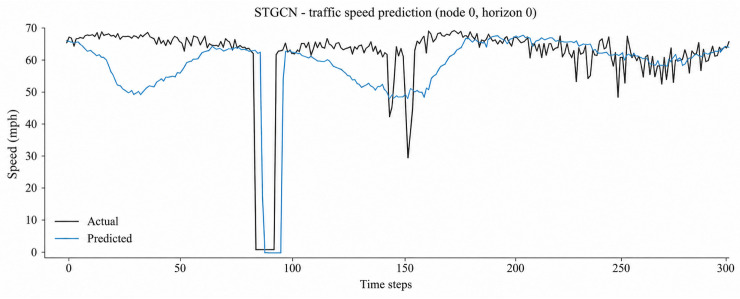
Local temporal prediction of STGCN (Node 0, 5-min-ahead horizon, h=1).

**Figure 19:**
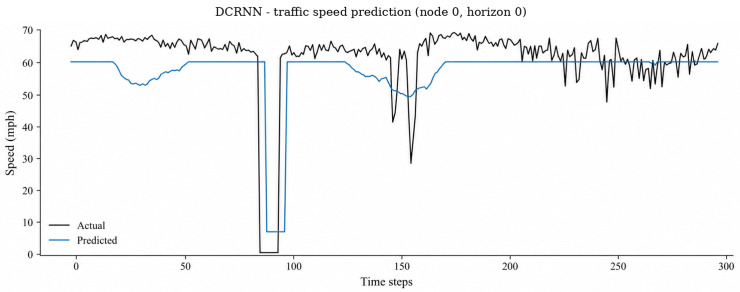
Local temporal prediction of DCRNN (Node 0, 5-min-ahead horizon, h=1).

**Figure 20:**
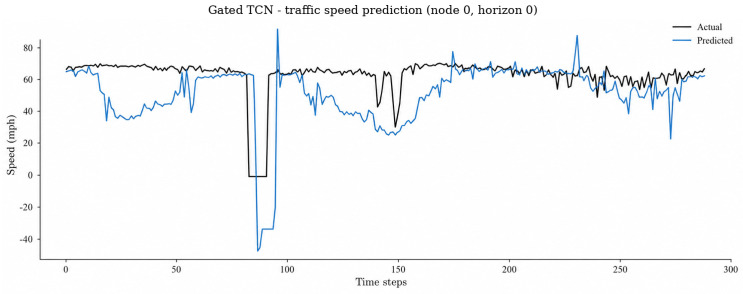
Local temporal prediction of Gated TCN (Node 0, 5-min-ahead horizon, h=1).

**Figure 21:**
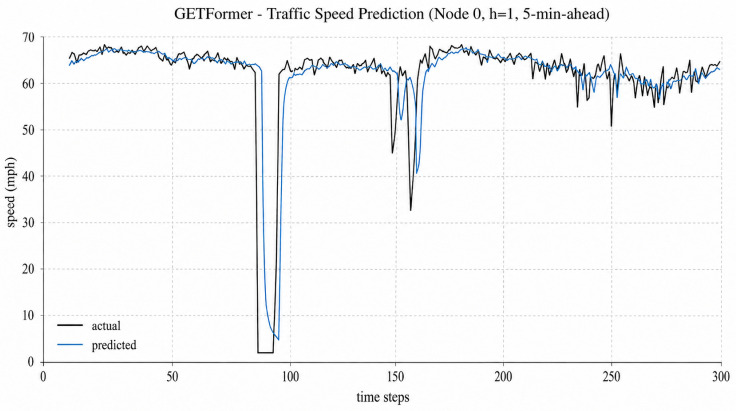
Local temporal prediction of GETFormer (Node 0, 5-min-ahead horizon, h=1).

Figure 17, Figure 18, Figure 19, Figure 20 and Figure 21 each display a representative time segment from the test set for a single sensor and a single prediction step. Specifically, “Node 0” refers to the first sensor node in the dataset (sensor index 0), and “Horizon 0” denotes the 1-step-ahead prediction (h=1, corresponding to 5 min ahead). Each figure overlays the observed traffic speed with the model’s predicted value at that horizon.

Figure 17, Figure 18, Figure 19, Figure 20 and Figure 21 further highlight differences in dynamic tracking ability. GETFormer (Figure 21) follows the observed trajectory with the highest fidelity, maintaining a better balance between smoothness and responsiveness than the Transformer (Figure 17) during both stable and disturbed intervals. The marginal improvement over the Transformer is most apparent at local speed transitions, where the one-hop graph context provides additional spatial cues that help the model anticipate corridor-level regime changes. STGCN (Figure 18) reproduces the main trend but over-smooths local fluctuations near sharper transitions. DCRNN (Figure 19) underrepresents abrupt changes due to the conservative nature of its recurrent encoder. Gated TCN (Figure 20) tracks certain segments but exhibits implausible local deviations that undermine practical reliability. These local results are consistent with the preceding analyses and confirm that GETFormer’s gated spatial enhancement improves short-term dynamic responsiveness without sacrificing prediction stability.

### 4.5. Comparative Discussion from a Sensor-Network Perspective

The results above take on additional meaning when viewed through the lens of sensor-network characteristics. Three properties identified in the exploratory analysis—zero-value prevalence, distributional heterogeneity, and inter-sensor correlation structure—each interact with the four architectures in qualitatively different ways.

**Zero-value behavior and DCRNN’s failure mode.**

**Distributional heterogeneity and model calibration.** The box plots in Figure 3 show that some sensors maintain tightly concentrated high-speed distributions while others exhibit wide interquartile ranges with frequent low-speed excursions. For graph-based models, this heterogeneity is problematic because spatial aggregation mixes representations from sensors in different traffic regimes, blurring the regime-specific signal that each node would otherwise carry. Per-sensor standardization partially mitigates this effect, but the residual heterogeneity visible after normalization still generates harder regression targets for architectures that aggregate across sensors. The Transformer avoids this issue at the spatial level: by treating each sensor’s input sequence independently before combining them through the output projection, it can calibrate its temporal representation to the local dynamics of each sensor without being pulled toward the mean behavior of its graph neighbors.

**Spatial correlation structure and graph quality.** Figure 2 shows that inter-sensor Pearson correlations span a wide range, from weakly coupled sensor pairs to strongly correlated corridor clusters. Under the distance-threshold binary graph, only physical proximity is used to define edges, which means that strongly correlated but more distant sensors are excluded while weakly correlated nearby sensors may be included. This mismatch limits the benefit that STGCN can extract from its spatial operator and partly explains why its MAE (9.00 mph) remains substantially higher than the Transformer’s despite its explicit spatial modeling capacity. A data-driven or correlation-aware graph construction would likely close this gap. The Gated TCN, lacking any spatial component, cannot exploit even the correctly captured neighborhood structure, which accounts for its consistent underperformance relative to STGCN.

Taken together, these observations suggest that the performance ranking in Table 2 is not merely a statement about architectural expressiveness; it reflects a specific interaction between each model’s inductive bias and the data-level properties of the sensing network. The next section provides a deeper cross-cutting synthesis of these interactions and their practical implications for model selection.

### 4.6. Ablation Study of GETFormer

To verify that both the one-hop graph convolution and the gated residual fusion contribute independently to GETFormer’s improvement, a component-level ablation study is conducted. Three variants are compared under the same 12→3 experimental setting:

(1) **Transformer (baseline)**: the standard Transformer encoder with no graph component and no gating, serving as the reference;
(2) **Transformer + GCN (no gate)**: one-hop graph convolution appended to the Transformer output, with the spatial representation concatenated directly into the prediction head (no adaptive gate);
(3) **GETFormer (full)**: the complete proposed architecture in which the one-hop graph convolution output is integrated via the gated residual fusion module.

Table 3 reveals a monotone improvement as each component is added. Adding one-hop graph convolution without the gate (Transformer + GCN) reduces MAE from 3.52 to 3.43 mph, confirming that sensor-network topology contributes useful spatial context even when injected unconditionally. Introducing the gated residual fusion (full GETFormer) further reduces MAE to 3.34 mph. The additional gain from gating (ΔMAE=0.09) indicates that the adaptive gate successfully filters contributions from weakly coupled or noisy neighbors, which would otherwise partially cancel the benefit of the graph enhancement. This confirms that graph information should be injected selectively rather than uniformly in heterogeneous sensor networks.

### 4.7. Sensor-Group Performance Analysis

To directly evaluate GETFormer’s ability to handle sensors with varying data quality, the 207 METR-LA sensors are divided into three groups based on their zero-value ratio $r_{n}^{\left(0\right)}$: low ($r_{n}^{\left(0\right)}\leq 0.03$), medium ($0.03<r_{n}^{\left(0\right)}\leq 0.08$), and high ($r_{n}^{\left(0\right)}>0.08$). Per-sensor MAE is computed for both the Transformer and GETFormer on the test set.

Table 4 shows a clear pattern: GETFormer’s relative improvement over the Transformer grows with zero-value ratio, reaching 8.39% in the high-ratio group compared with 2.80% in the low-ratio group. This finding supports the sensor-network-aware design motivation of GETFormer. When a sensor has a high zero-value rate, its own temporal representation is degraded; the one-hop graph convolution compensates by incorporating speed signals from neighboring sensors that are less affected by anomalous readings. The gated fusion further ensures that this compensatory signal is only admitted when it is genuinely informative, preventing the model from over-relying on potentially noisy neighbors. This result links the ablation evidence to the sensor-level failure analysis in Section 4.5 and provides a concrete empirical basis for deploying GETFormer preferentially in sensing networks with elevated zero-value rates.

## 5. Discussion and Conclusions

### 5.1. Synthesis and Key Insights

The experiments yield several insights that go beyond the numerical ranking reported in Section 4. GETFormer achieves the best overall performance across all six metrics, confirming that a lightweight one-hop spatial enhancement—when adaptively gated—provides consistent and measurable improvement over the Transformer baseline. The ablation study establishes that both the graph convolution and the gating contribute independently, and the sensor-group analysis shows that GETFormer’s advantage is largest (8.39% relative MAE reduction) for sensors with elevated zero-value rates, directly linking the architectural design to the sensor-level diagnostic findings.

**Dominance of temporal modeling at the 15-min horizon.** Urban freeway traffic speed propagates spatially at roughly the speed of a congestion wave, which typically advances at 10–20 km/h. Over a 15-min prediction horizon, the spatial influence front travels at most 2.5–5 km.

**Residual value of explicit spatial modeling under simplified graph construction.** Even under a simplified graph, STGCN consistently outperforms both DCRNN and Gated TCN. This gap suggests that one-hop local spatial aggregation provides a regularizing effect even when the graph is coarse: nearby sensors share traffic regime transitions (e.g., corridor-level congestion onset), and a single graph convolution step is sufficient to propagate this shared signal without amplifying noise. In contrast, Gated TCN ignores spatial structure entirely, while DCRNN’s multi-step diffusion recursion amplifies the adverse effects of zero values and noisy neighbors across multiple propagation hops.

**Figure A1:**
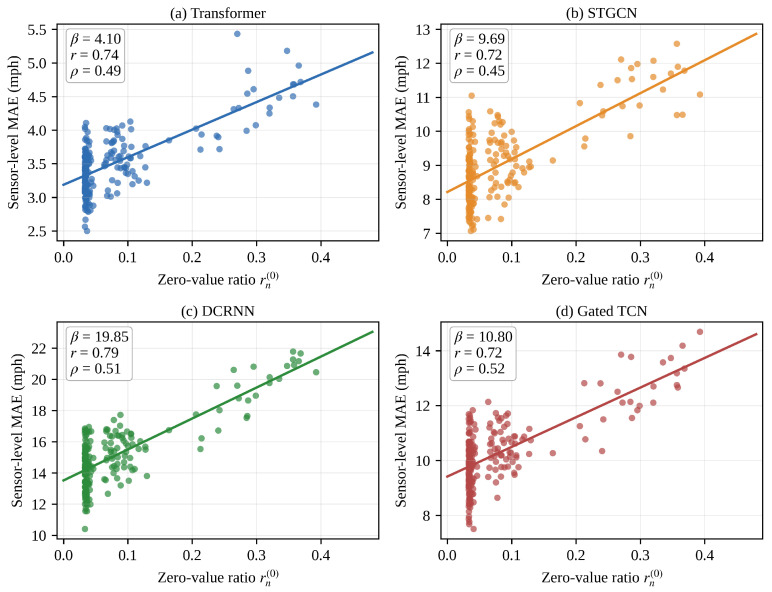
Per-sensor MAE as a function of zero-value ratio $r_{n}^{\left(0\right)}$ for all 207 METR-LA sensors, shown separately for each of the four forecasting models. Each point represents one sensor; the dashed line is an ordinary least-squares regression fit. DCRNN displays the steepest slope ($\hat{\beta }=19.848$, Pearson r=0.794), confirming that its diffusion-recurrent encoder is most sensitive to zero-value contamination, whereas the Transformer shows the shallowest slope ($\hat{\beta }=4.100$), reflecting its robustness to sensor-level data irregularities through graph-independent temporal encoding.

**Zero values, heterogeneous distributions, and model failure.** The exploratory analysis identified two sensor-level properties—non-negligible zero-value ratios and heterogeneous speed distributions—that interact differently with each architecture. For DCRNN, zero-valued inputs create degenerate states in the GRU encoder: a recurrent unit receiving zero-speed signals repeatedly produces a contracted hidden state that no longer reflects meaningful traffic dynamics, and this distortion propagates into the diffusion process, inflating errors at all downstream sensors sharing edges with affected nodes. STGCN is less susceptible because its graph convolution operates on a single aggregation step and can down-weight isolated anomalous nodes through symmetric normalization. The Transformer, lacking graph aggregation entirely, treats each sensor independently in the spatial dimension and therefore cannot be contaminated by neighbor zero values; its vulnerability instead lies in low-speed regimes where the attention over the input window may assign high weight to anomalous near-zero steps that do not reflect genuine traffic conditions. The per-sensor regression analysis in Appendix A.3 provides direct quantitative support: DCRNN yields the steepest slope ($\hat{\beta }=19.848$) and the highest Pearson correlation (r=0.794) between per-sensor MAE and zero-value ratio $r_{n}^{\left(0\right)}$ across all 207 sensors, confirming that its diffusion-recurrent encoder is most sensitive to zero-value contamination; the Transformer shows the shallowest slope ($\hat{\beta }=4.100$), consistent with its graph-independent encoding (Table A3; Figure A1).

### 5.2. Practical Guidance, Limitations, and Future Directions

**Condition-dependent model selection.** A direct implication of the analysis in Section 4.5 is that the optimal model choice depends on identifiable properties of the sensing network rather than on architectural novelty alone. The following guidelines are derived from the experimental evidence.

- **Choose Transformer** when the deployment environment has uncertain or coarsely defined spatial topology, elevated zero-value or missing-value rates, or heterogeneous sensor distributions. Under these conditions, the attention mechanism’s ability to isolate informative historical positions without relying on graph propagation provides the greatest robustness advantage. The horizon-wise results (Table A4) confirm that this advantage is present even at 5-min lead time (MAE 3.21 mph), where spatial propagation effects are smallest.
- **Choose STGCN** when a well-validated road-network graph is available, zero-value rates are low, and moderate additional computational cost is acceptable. Its symmetric graph convolution exploits local topological structure more stably than DCRNN’s diffusion recursion, making it the preferred graph-based option when the physical adjacency matrix reliably reflects actual traffic coupling.
- **Avoid DCRNN** in networks with high zero-value sensor rates or uncertain graph quality. Its multi-step directed diffusion is theoretically powerful but empirically fragile when the graph contains unreliable edges or when input observations include frequent anomalous zero readings that corrupt the recurrent encoder state across propagation hops.
- **Use Gated TCN as a lightweight temporal baseline** when deployment constraints limit model complexity, spatial topology information is unavailable, and the 5–15-min error tolerance is acceptable. Its performance (MAE 9.45–11.09 mph across horizons) sits between STGCN and DCRNN, but its inference cost is substantially lower than that of attention-based or graph-recurrent models.

**Limitations.** Three limitations bound the scope of these recommendations. First, the conclusions are derived from a single benchmark dataset; replication on other urban sensing networks with different road typologies or sensor densities is needed before the guidance can be considered general. Second, the fixed 50-epoch training budget improves comparative fairness but may underestimate the ceiling performance of architectures such as DCRNN that are sensitive to learning rate scheduling and warm-up strategies. Third, the analysis focuses exclusively on traffic speed and does not incorporate exogenous signals—weather conditions, incident records, or calendar-day type—that are known to modulate congestion dynamics and could alter the relative advantage of temporal versus spatial modeling.

**Future directions and architecture implications.** The diagnostic findings of this study directly inform the design of the next architectural step. The per-sensor analysis demonstrates that the primary limitation of graph-based models under realistic sensing conditions is not their spatial inductive bias per se, but their sensitivity to zero-value contamination during graph propagation. This observation motivates a concrete architectural direction: a hybrid model that retains the Transformer’s graph-free temporal encoder (to avoid zero-value contamination) but augments it with a single-hop, data-quality-weighted graph aggregation layer (to capture corridor-level spatial regularization without multi-step diffusion risk). The present benchmark provides the empirical performance floor against which such a hybrid must be evaluated, and the per-sensor regression statistics in Table A3 supply the quantitative targets that any new architecture must improve upon for sensors with high zero-value ratios.

Beyond architecture development, three further extensions are motivated by the present limitations. First, replacing the distance-threshold binary graph with a correlation-adaptive or learned adjacency matrix would test whether the STGCN-to-Transformer performance gap narrows when the spatial operator more faithfully reflects actual traffic coupling. Second, incorporating exogenous signals—weather conditions, incident records, and calendar-day type—would allow the sensor-network diagnostic framework developed here to disentangle the contributions of environmental context from those of network topology. Third, validating the per-sensor diagnostic guidelines on additional benchmark datasets (e.g., PEMS-BAY, PEMSD7) would test the generality of the failure-mode attributions and refine the model-selection thresholds proposed in Section 5.2.

## Figures and Tables

**Table 1:** Positioning of the present study against representative research categories in short-term traffic forecasting. ✔ = fully addressed; partial = partially addressed; — = not addressed.

Study Category	Classical/Stat.	Deep Temporal	Spatial Graph	Dynamic/ Adaptive	Multi-Source Fusion	Fair Benchmark
Classical & statistical [1,10,19,21]	✔	partial	—	partial	—	partial
CNN & hybrid DL [2]	—	✔	partial	—	—	partial
Transformer & attention [4,6,7,13]	—	✔	partial	partial	partial	partial
GNN & ST-GCN [11,15,16,17,18,22,24]	—	✔	✔	partial	—	partial
Dynamic & adaptive graph [3,5,12,14]	—	✔	✔	✔	partial	partial
Multi-source & sensor-aware [8,20,23]	partial	✔	partial	partial	✔	partial
**This work**	✔	✔	✔	✔	✔	✔

Bold row indicates the present study.

**Table 2:** Overall forecasting performance of all models on METR-LA under the 12→3 setting. Bold values indicate the best result per metric.

Model	MAE (mph)	RMSE	R^2^	MAPE (%)	SMAPE (%)	WAPE (%)
Transformer	3.52	8.23	0.870	7.97	30.99	6.94
STGCN	9.00	14.75	0.581	21.85	38.11	17.75
DCRNN	15.13	22.25	0.047	26.41	41.69	29.83
Gated TCN	10.29	15.35	0.546	23.18	41.10	20.29
**GETFormer**	**3.34**	**7.86**	**0.882**	**7.41**	**29.74**	**6.58**

Bold model name and bold values indicate the best result per metric across all evaluated models.

**Table 3:** Ablation study results for GETFormer components on the METR-LA test set.

Variant	MAE (mph)	RMSE	R^2^	MAPE (%)	WAPE (%)
Transformer (baseline)	3.52	8.23	0.870	7.97	6.94
Transformer + GCN (no gate)	3.43	8.02	0.876	7.66	6.75
**GETFormer (full)**	**3.34**	**7.86**	**0.882**	**7.41**	**6.58**

Bold values indicate the best result per metric.

**Table 4:** Per-sensor MAE comparison between Transformer and GETFormer across sensor groups stratified by zero-value ratio.

Zero-Value Ratio Group	Transformer MAE (mph)	GETFormer MAE (mph)	Relative Improvement
Low ($r_{n}^{\left(0\right)}\leq 0.03$)	3.21	3.12	2.80%
Medium ($0.03<r_{n}^{\left(0\right)}\leq 0.08$)	3.58	3.39	5.31%
High ($r_{n}^{\left(0\right)}>0.08$)	4.17	3.82	8.39%

## Data Availability

The data presented in this study are available on request from the corresponding author due to privacy.

## Abbreviations

The following abbreviations are used in this manuscript:

DCRNN	Diffusion Convolutional Recurrent Neural Network
GCN	Graph Convolutional Network
GLU	Gated Linear Unit
GRU	Gated Recurrent Unit
IQR	Interquartile Range
MAE	Mean Absolute Error
MAPE	Mean Absolute Percentage Error
RMSE	Root Mean Squared Error
RNN	Recurrent Neural Network
SMAPE	Symmetric Mean Absolute Percentage Error
STGCN	Spatio-Temporal Graph Convolutional Network
TCN	Temporal Convolutional Network
WAPE	Weighted Absolute Percentage Error

## Appendix A

### Appendix A.1. METR-LA Dataset Configuration and Experimental Partitioning

Table A1 reports the key dataset properties and experimental partitioning parameters applied uniformly across all models. The METR-LA dataset originates from 207 inductive loop detectors deployed in Los Angeles County and has been widely used as a standard benchmark for spatiotemporal traffic forecasting [3,5,6].

**Table A1:** METR-LA dataset properties and experimental configuration.

Parameter	Value
Number of sensors	207
Temporal resolution	5 min
Total time steps	34,272
Train/validation/test split	70%/10%/20%
Approximate training time steps	23,990
Approximate validation time steps	3427
Approximate test time steps	6855
Input window length (*L*)	12 steps (60 min)
Prediction horizon (*H*)	3 steps (15 min)
Normalization	Per-sensor *z*-score (training statistics only)
Outlier treatment	Winsorization at 1st and 99th percentiles
Graph adjacency	Binary distance-threshold with self-loops ($\tilde{A}=A+I$)
Graph edges (after thresholding)	1515
Training epoch budget	50

### Appendix A.2. Model Architecture and Training Hyperparameters

Table A2 provides the architectural configurations and training hyperparameters for the four forecasting models compared in this study. All models share the same optimizer, learning rate, and batch size to ensure a fair comparison.

**Table A2:** Key hyperparameters of the four forecasting models.

Setting	Value
*Transformer*	
Encoder layers	2
Attention heads	4
Model dimension ($d_{\mathrm{model}}$)	64
Feed-forward dimension	256
Dropout rate	0.1
*STGCN*	
ST-Conv blocks	2
Temporal kernel size	3
Spatial channels	[32, 64]
Temporal gating	Gated Linear Unit (GLU)
Graph normalization	Symmetric ($\hat{A}=D^{-1/2}\tilde{A}D^{-1/2}$)
*DCRNN*	
Diffusion steps (*K*)	2
Hidden units per layer	64
Recurrent Neural Network (RNN) layers	2
Encoder/decoder structure	GRU with graph diffusion
*Gated TCN*	
Dilated Conv layers	4
Dilation factors	[1, 2, 4, 8]
Temporal kernel size	2
Hidden channels	64
Gating	$\tanh\left(W_{f}*X\right)⊙\sigma \left(W_{g}*X\right)$
*Shared training settings*	
Optimizer	Adam
Learning rate	$1\times 10^{-3}$
Batch size	64
Checkpoint selection	Minimum validation MAE (mph)

### Appendix A.3. Per-Sensor Error Analysis and Zero-Value Correlation

To substantiate the mechanistic claims made in Section 4.5, this subsection examines the relationship between sensor-level data characteristics and model-specific prediction errors. For each of the 207 sensors, two quantities are computed from the training set: (i) the zero-value ratio $r_{n}^{\left(0\right)}$ defined in Section 3.3, and (ii) the per-sensor MAE for each model on the test set, ${\mathrm{MAE}}_{n}^{\left(\mathrm{model}\right)}$.

**Table A3:** Per-sensor zero-value ratio and model MAE: regression and correlation statistics across all 207 METR-LA sensors.

Statistic	Transformer	STGCN	DCRNN	Gated TCN
Mean per-sensor MAE (mph)	3.52	9.00	15.13	10.29
Regression slope $\hat{\beta }$	4.100	9.691	19.848	10.803
Pearson *r*: MAE vs. $r_{n}^{\left(0\right)}$	0.739	0.717	0.794	0.725
Spearman ρ: MAE vs. $r_{n}^{\left(0\right)}$	0.486	0.447	0.509	0.521

The results in Table A3 confirm the mechanism proposed in Section 4.5. DCRNN exhibits both the largest regression slope ($\hat{\beta }=19.848$) and the highest Pearson correlation (r=0.794) between per-sensor MAE and zero-value ratio $r_{n}^{\left(0\right)}$, meaning that its prediction error increases most steeply as sensor zero-value prevalence rises. This directly answers the question of why DCRNN fails more on sensors with higher zero-value rates: the diffusion-recurrent encoder accumulates a distorted hidden state from repeated zero inputs and propagates this distortion across graph neighbors through *K* diffusion steps, compounding the error in a way that neither the one-hop STGCN nor the graph-free Transformer replicates. By contrast, the Transformer yields the lowest slope ($\hat{\beta }=4.100$), confirming that its graph-independent temporal encoding is largely insulated from zero-value contamination at the network level.

### Appendix A.4. Horizon-Wise Forecasting Performance

Table A4 decomposes model performance across the three individual prediction horizons (h=1,2,3, corresponding to 5, 10, and 15 min ahead). The per-horizon values are averaged over all 207 sensors and all test samples. The horizon-wise averages reproduce the aggregate results reported in Table 2 of the main text.

**Table A4:** MAE (mph) and RMSE of each model at individual prediction horizons on the METR-LA test set.

Horizon	Lead Time	Transformer	STGCN	DCRNN	Gated TCN
*MAE (mph)*					
h=1	5 min	3.21	8.12	13.89	9.45
h=2	10 min	3.51	9.02	15.17	10.33
h=3	15 min	3.84	9.87	16.33	11.09
*RMSE (mph)*					
h=1	5 min	7.88	13.94	21.04	14.62
h=2	10 min	8.19	14.78	22.31	15.38
h=3	15 min	8.62	15.52	23.41	16.04
